# Immune activation during liver machine perfusion is shaped by perfusate composition and is independent of the ischemic injury

**DOI:** 10.3389/fimmu.2026.1874888

**Published:** 2026-09-14

**Authors:** Ida H. Færden, Marte Bliksøen, Camilla Schjalm, Kristin Pettersen, Olav M. I. B. Liavåg, Waleed M. Majeed, Morten Hagness, Tom E. Mollnes, Søren E. Pischke

**Affiliations:** 1 Department of Immunology, Oslo University Hospital Rikshospitalet, Oslo, Norway; 2 Institute of Clinical Medicine, University of Oslo, Oslo, Norway; 3 Division of Emergencies and Critical Care, Oslo University Hospital Rikshospitalet, Oslo, Norway; 4 Section for Transplantation Surgery, Department of Transplantation Medicine, Oslo University Hospital, Oslo, Norway; 5 Research Laboratory, Nordland Hospital, Bodø, Norway

**Keywords:** complement activation, cytokine production and release, innate immunity, liver machine perfusion, liver transplantation

## Abstract

**Background:**

Ischemia–reperfusion injury remains a major challenge in liver transplantation and plays a major role in pathophysiology leading to graft failure. Machine perfusion (MP) is a promising approach to restitute marginal donor livers. However, the impact of MP on innate immunity remains insufficiently defined. We hypothesized that MP activates the innate inflammatory response. Thus, we investigated whether the ischemic injury and perfusate composition [plasma-rich perfusate (PRP) versus plasma-poor perfusate (PPP)] modulate the inflammatory response, identified by complement activation and cytokine release.

**Methods:**

Thirty porcine livers were subjected to *ex situ* MP. The first 24 porcine livers were subject to induced biliary ischemic injury (BileINJ, *n* = 8), global ischemic injury (GlobalINJ, *n* = 8), or no induced ischemic injury (CTRL, *n* = 8) using PRP during MP. The subsequent six porcine livers were subjected to the same *ex situ* MP protocol using PPP (*n* = 6) with no induced ischemic injury. MP was conducted in three phases: 1 h hypothermic, 1 h controlled rewarming, and 4 h normothermic perfusion.

**Results:**

Except for interleukin-1β (IL-1β) in bile tissue in the BileINJ group and tumor necrosis factor (TNF) in plasma in the GlobalINJ group, all mediators assessed whether in perfusate, liver, or bile tissue did not differ significantly between ischemic injury groups and CTRL. Data were therefore combined into a common PRP group for further comparisons. In PRP perfusate, MP induced robust complement activation (sC5b-9: median fold change (FC) 4 [2–6 interquartile range]) and cytokine release (IL-1β: FC 88 [51–165], IL-6: FC 1,549 [587–3,053], IL-8: FC 389 [120–1,259], IL-10: FC 404 [222–656], all *p* < 0.001). This pattern persisted in PPP group perfusate, liver, and bile tissue, though the concentrations were significantly lower compared to PRP (sC5b-9, IL-10, IL-6, IL-1β, and IL-8, all *p* < 0.05).

**Conclusions:**

Liver MP reliably induced innate immune-driven inflammation regardless of prior ischemic injury. The magnitude of the inflammatory response was substantially lower in the PPP group compared to the PRP group. Future therapeutic trials targeting innate immunity during *ex situ* MP are warranted to improve the preservation of liver grafts.

## 1 Introduction

Ischemia–reperfusion injury (IRI) remains a significant barrier to successful liver transplantation by contributing to the pathophysiology of short- and long-term graft failure (1, 2). IRI is thought to be mediated through activation of the innate immune system, particularly the complement system (1, 2). To address organ shortages, increasing utilization of extended criteria donors and donations after circulatory death further increases susceptibility to IRI. Machine perfusion (MP) has demonstrated potential for reducing IRI and increasing organ utilization (3, 4). However, despite promising results with up to 20% otherwise discarded livers found viable after MP, many grafts still fail to regain function during MP (4). It has been hypothesized that this may be attributed to the inflammatory responses during MP that may impact graft function, yet the mechanistic role of innate immunity in this context remains less defined.

The complement system is a key component of innate immunity, essential for recognition of danger by pathogens and damaged cells (5, 6). Its involvement in IRI has been studied extensively, revealing its critical function in the propagation of liver IRI (1, 7–9). Its three activation pathways—the classical, lectin, and alternative pathways—converge at the level of C3, ultimately cleaving C5 to the potent anaphylatoxin C5a and the terminal complement complex (TCC), either inserted in a membrane as C5b-9 (membrane attack complex) or released in its soluble form (sC5b-9).

In transplantation and IRI, the lectin and alternative pathways are particularly activated (2, 10). Complement activation is well documented in extracorporeal systems such as extracorporeal membrane oxygenation (11–13), cardiopulmonary bypass (14, 15), and hemodialysis (16). Similarly, normothermic MP (NMP) of kidneys induces complement activation and cytokine release (17, 18); however, comparable data in liver MP remain limited.

Given the pivotal role of the complement system in IRI, modulating its activity during MP could enhance graft preservation and function (2). Therefore, our primary aim was to investigate complement activation and downstream cytokine release in porcine livers subjected to different ischemic insults. Our secondary objective was to evaluate the influence of perfusate composition on immune activation during MP, identifying potential strategies to reduce immune activation and improve liver transplant outcomes. We previously used biosensors to evaluate viability of ischemic injured porcine livers during *ex situ* MP (19). Here, we extend that work by examining innate immune activation, particularly the complement system and the cytokine network, during *ex situ* MP of the same livers.

## 2 Methods

### 2.1 Animals and experimental design

This sub-study builds on the previously published study (19), including 24 Norwegian Landrace pigs (*Sus scrofa domesticus*, body weight 60 kg ±10%, 9 females, 14 males, and 1 missing data, liver weight 1.59 ± 0.25 kg). Six additional pigs (one female and five males, liver weight 1.62 ± 0.33 kg) were included for this sub-study and subjected to the same procedure as in the main study with the exception of the plasma-rich perfusate (PRP) used during rewarming and NMP being replaced with plasma-poor perfusate (PPP) (**Figure 1**). All pigs included in this study were approved by the Norwegian Food Safety Authority (FOTS 24454), and the study was conducted in accordance with national animal welfare and the ARRIVE guidelines.

**Figure 1:**
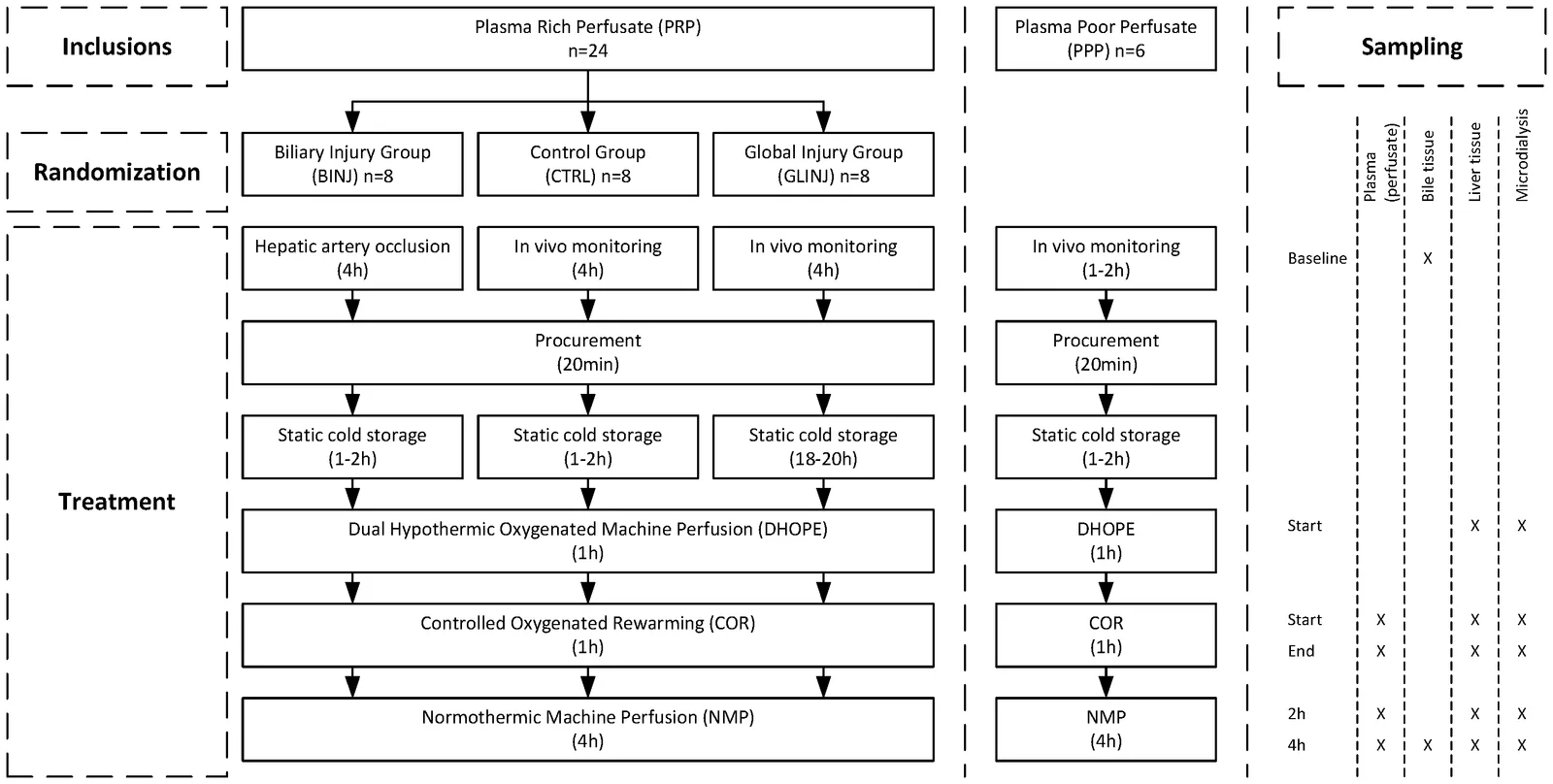
Study design overview.

The detailed surgical and experimental procedure has recently been described (19). Briefly, all livers experienced a total warm ischemia time of approximately 20 min in relation to the procurement procedure. All livers were flushed with ice-cold Institute Georges Lopez-1 solution (IGL-1) (IGL, IGL group, France) after cross-clamp. The 24 pigs where PRP was used as perfusate were divided into three randomized groups to assess ischemic injury and complement activation during MP: Control (CTRL), Biliary Injury (BileINJ), and Global Liver Injury (GlobalINJ). Histopathological evaluations confirmed the group-specific induced ischemic liver and bile injuries (19). The CTRL group was subjected to 4 h of *in vivo* monitoring, followed by liver procurement and 1–2 h of static cold storage (SCS) preserved with IGL-1. The BileINJ group was subjected to 4 h of hepatic artery occlusion *in vivo*, followed by liver procurement and 1–2 h of SCS preserved with IGL-1. The HA was occluded using plastic clips and 5,000 IE of heparin was injected intravenously 5 min prior to occlusion. The GlobalINJ group was subjected to 4 h of *in vivo* monitoring, followed by liver procurement and a prolonged period of 18–20 h of SCS preserved with IGL-1. The additional six pigs, where PPP was used as perfusate, were subjected to 1–2 h of *in vivo* monitoring, followed by liver procurement and 1–2 h of SCS preserved with IGL-1. Subsequently, all livers underwent *ex situ* MP using the Liver Assist device (XVIVO, Gothenburg, Sweden) according to the Groningen protocol (20). This protocol is structured into three phases: 1 h of dual hypothermic machine perfusion (DHOPE) at 8–10°C, 1 h of controlled oxygenated rewarming (COR) to reach porcine normothermia of 38˚C, and 4 h of NMP at 38°C. Pre-cooled Belzer Machine Perfusion Solution (Bridge to Life Ltd, Wandsworth, UK) was used as perfusate during hypothermic MP. For the PRP group, heparinized (525 IU/kg pig weight) leukocyte- and thrombocyte-depleted autologous whole blood was used during rewarming and NMP. Leukocyte and thrombocyte depletion was achieved using a filter according to manufacturer’s instruction (Fresenius-Kabi BioR Flex, Hamburg Germany). For the PPP group, the whole blood was leukocyte- and thrombocyte-depleted as described above. Thereafter, depleted whole blood was centrifuged twice (2,000 × *g* at 20°C for 20 min) and washed with phosphate-buffered saline (800 mL) leaving only red blood cells [**Supplementary Table 1** (PRP) and 2 (PPP)].

Complement and cytokines were measured in the autologous plasma both *in vivo* at baseline and prior to procurement for both groups. No significant differences in the autologous plasma were seen between the groups prior to MP (**Supplementary Figure 1**). Complement activation was assessed in the process of leukocyte and platelet filtration (Fresenius-Kabi BioR Flex) of whole blood (PRP *n* = 6) and in the process of plasma washout (PPP *n* = 2). Leukocyte and platelet depletion of porcine whole blood significantly increased complement activation (sC5b-9) compared to non-filtrated whole blood (*p* < 0.002) (**Supplementary Figure 2A**). Plasma washout in the PPP process reduced sC5b-9 levels to baseline levels (**Supplementary Figure 2B**).

### 2.2 Sample collection and placement of biosensors

Perfusate samples were collected at pre-defined timepoints during rewarming and NMP in tubes with 0.2 M EDTA final concentration, directly placed on crushed ice, and centrifuged at 3,000 × *g* for 15 min at 4°C (**Figure 1**). Supernatant (plasma) was allocated and frozen immediately at −80°C for later analysis. Biopsies of liver parenchyma were collected at start hypothermic MP and at pre-defined timepoints during rewarming and NMP. Bile duct biopsies were collected at baseline *in vivo* and at 4 h NMP due to limited tissue for sampling. All tissue biopsies were frozen at −80°C for later analysis.

Five microdialysis catheters (M Dialysis, Solna, Sweden) were inserted into the liver tissue (*n* = 2), the common bile duct (*n* = 2), and hilar plate (*n* = 1). For detailed description of the methodology, reference is made to the main study (19). Briefly, equilibration of metabolic and inflammatory biomarkers occurs through a semipermeable membrane at the tip of the catheter [CMA 70, pore size 100 kDa, length 30 mm (liver tissue and bile duct) and 10 mm (hilar plate), M Dialysis, Solna, SWE] with the steady flow of dialysis fluid [hydroxyethyl starch 130/0.4 (Voluven^®^, Hospira, Lake Forest, IL)] inside the catheter. The dialysis fluid was continuously pumped at 1 μL/min into a collection tube (Microvial, M Dialysis). The microvial was collected at pre-defined timepoints, directly placed on crushed ice, and analyzed for metabolic substances prior to being frozen at −80°C for later C3a analysis (**Figure 1**).

### 2.3 Immunoassays

In-house enzyme-linked immunosorbent assays (ELISAs) were used to measure C3a in EDTA perfusate and microdialysate, and soluble C5b-9 (sC5b-9) in EDTA perfusate, liver, and bile duct tissue, as previously described (21, 22), and modified for pig (23). Cytokines in perfusate and tissue were measured using commercially available assays previously assessed for porcine specificity (24). Commercial ELISAs were used to detect tumor necrosis factor (TNF) and interleukin-1β (IL-1β) (whole protein tissue extract) and IL-6 (EDTA perfusate) (R&D Systems, Minneapolis, MN). Multiplex assays were used for assessment of IL-8 and IL-6 (whole protein tissue extract) (R&D Systems), IL-10 (Invitrogen, Carlsbad, CA), and IL-8 and IL-1β (EDTA perfusate) (Merck Millipore, Darmstadt, Germany). All assays were performed according to the manufacturer’s instructions. Tissue extraction was performed using CytoBuster protein extraction reagent (Millipore Sigma, Billerica, MA) and complete protease inhibitor cocktail (Roche, Basel, Switzerland) as previously described (24).

### 2.4 Statistical analysis

Statistical analysis using GraphPad Prism 10 (GraphPad Software, San Diego, CA) was performed to assess innate immune system activation through complement activation and cytokine production during liver MP. Fold change (FC) values were calculated comparing end NMP (6 h MP) to start NMP (2 h MP) for perfusate and for liver tissue. Because of limited material for tissue sampling of the bile duct, FC values for bile duct were calculated comparing *in vivo* baseline and end NMP.

Nonparametric analyses were chosen due to the small sample size and the exploratory nature of this study. Analyses were performed on all perfusate, tissue, and microdialysis samples. Mixed-effects models with assumed sphericity were used for comparisons between groups; (A) between the ischemic injured groups (BileINJ and GlobalINJ) and control (CTRL), and (B) between perfusate types (PRP or PPP). No *post-hoc* correction for multiple comparisons was performed. Wilcoxon matched-pairs signed-rank test was used for time comparisons. Data are presented as median (interquartile range). Statistical significance was determined at *p* < 0.05.

Overall, no significant differences were observed between the ischemic injury groups (BileINJ and GlobalINJ) and the control group (CTRL) during MP of PRP livers. Therefore, all PRP groups were combined for further comparisons with PPP except for IL-1β in BileINJ (bile tissue) and TNF in GlobalINJ (plasma), which were significantly different from CTRL.

## 3 Results

### 3.1 Complement activation during MP of ischemically injured livers

Perfusate, liver, and bile tissue sC5b-9, as well as perfusate and microdialysate C3a, did not differ between PRP groups and were thus combined in further analyses as described in Methods (**Figures 2A–F**). Perfusate sC5b-9 was significantly higher at end NMP (6 h MP) compared to start NMP (2 h MP, peak median FC 1.6 [1.4–3.1 interquartile range], both *p* < 0.001, **Figure 2A**). Similarly, bile tissue sC5b-9 was significantly elevated at 6 h MP compared to baseline (FC 1.4 [0.9–1.8], *p* = 0.004, **Figure 2C**). Perfusate C3a had no significant change during NMP in perfusate or microdialysate. However, perfusate C3a was significantly decreased at start NMP (2 h MP) compared to start rewarming (1 h MP, *p* < 0.001, **Figure 2D**). Hilar plate microdialysate C3a significantly increased at start and end NMP (2 and 6 h) compared to start hypothermic MP (0 h MP, both *p* < 0.001, **Figure 2E**). Bile microdialysate C3a significantly increased during rewarming (1 and 2 h MP) compared to start hypothermic MP (0 h MP, both *p* < 0.001, **Figure 2F**).

**Figure 2:**
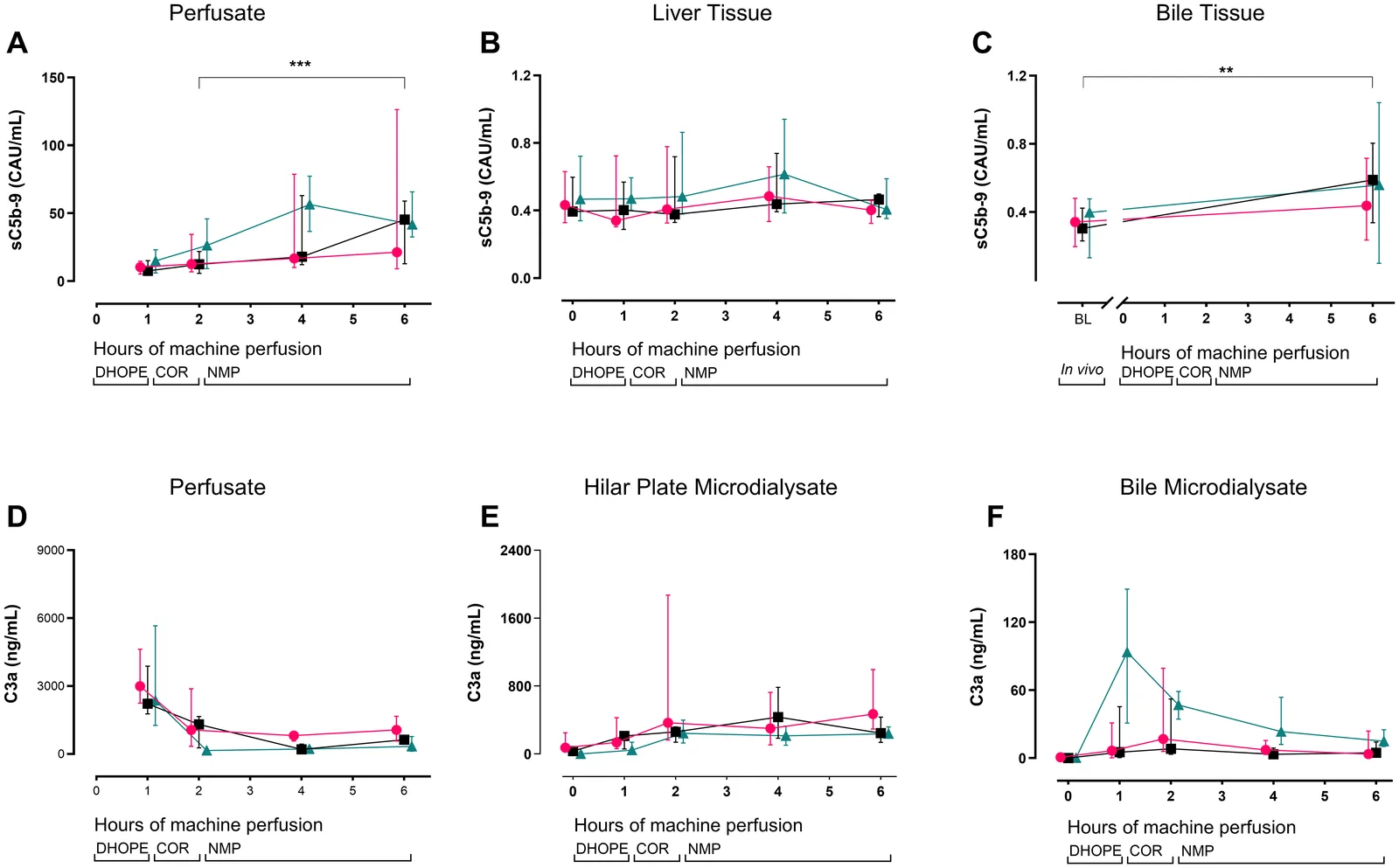
Complement activation in perfusate, liver, and bile tissue and hilar plate and bile microdialysate during liver machine perfusion using plasma-rich perfusate [**(A–C)**: sC5b-9, **(D–F)**: C3a]. BL, baseline; DHOPE, dual hypothermic oxygenated perfusion; COR, controlled oxygenated rewarming; NMP, normothermic machine perfusion. Values are presented as median; vertical whiskers present interquartile range. Horizontal lines with *p*-values present end normothermic MP (6 h MP) compared to start normothermic MP (2 h MP; perfusate, liver tissue, and microdialysates) and *in vivo* baseline (bile tissue), for CTRL alone or CTRL pooled with GlobalINJ and/or BileINJ when no significant difference between groups was identified. Colored asterisk presents BileINJ or GlobalINJ, respectively, compared to CTRL over time during MP. *p < 0.05, **p < 0.01, ***p < 0.001.

#### 3.1.1 Cytokine release during MP of ischemic injured livers

Perfusate cytokines IL-1β, IL-6, IL-8, and IL-10 did not differ between PRP groups and were thus combined in further analyses as described in Methods (**Figures 3A, D**, **4A, D**). All perfusate cytokines were significantly higher at end NMP (6 h MP) compared to start NMP (2 h MP, IL-1β: FC 44 [17–63], IL-6: FC 648 [207–1,545], IL-8: FC 216 [67–653], IL-10: FC 314 [105–527], all *p* < 0.001, **Figures 3A, D**, **4A, D**). Perfusate TNF in the GlobalINJ group was significantly lower during the whole course of MP compared to CTRL (*p* = 0.02, **Figure 3G**) but increased similarly and significantly at end NMP compared to start NMP in all groups (2 h MP, all *p* < 0.02, **Figure 3G**).

**Figure 3:**
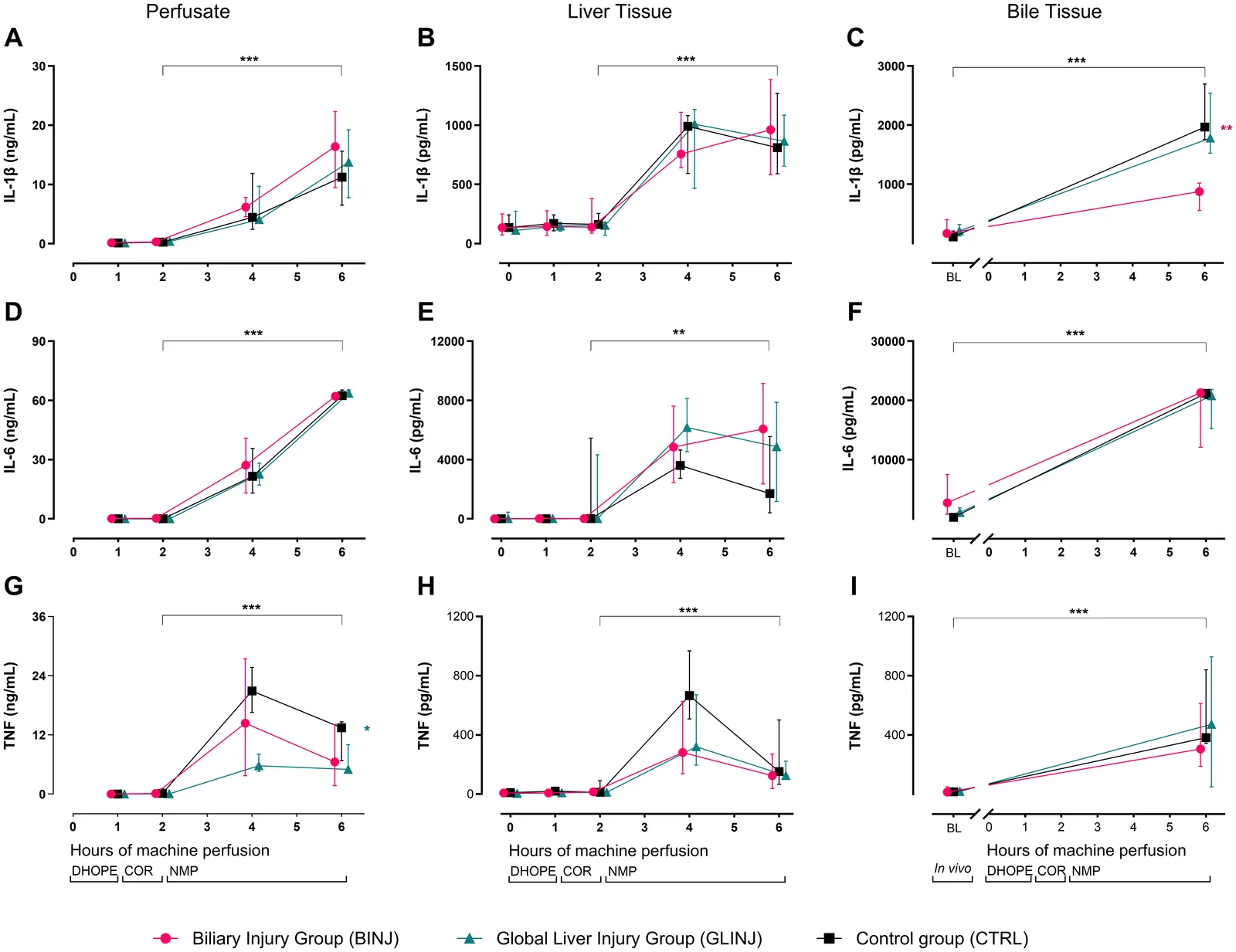
Proinflammatory cytokine concentrations in perfusate, liver, and bile tissue during liver machine perfusion using plasma-rich perfusate [**(A–C)**: IL-1β, **(D–F)**: IL-6, **(G–I)**: TNF]. BL, baseline; DHOPE, dual oxygenated hypothermic perfusion; COR, controlled oxygenated rewarming; NMP, normothermic machine perfusion. Values are presented as median; vertical whiskers present interquartile range. Horizontal lines with *p*-values present end normothermic MP (6 h MP) compared to start normothermic MP (2 h MP; perfusate and liver tissue) and *in vivo* baseline (bile tissue), for CTRL alone or CTRL pooled with GlobalINJ and/or BileINJ when no significant difference between groups was identified. Colored asterisk present BileINJ or GlobalINJ, respectively, compared to CTRL over time during MP. *p < 0.05, **p < 0.01, ***p < 0.001.

**Figure 4:**
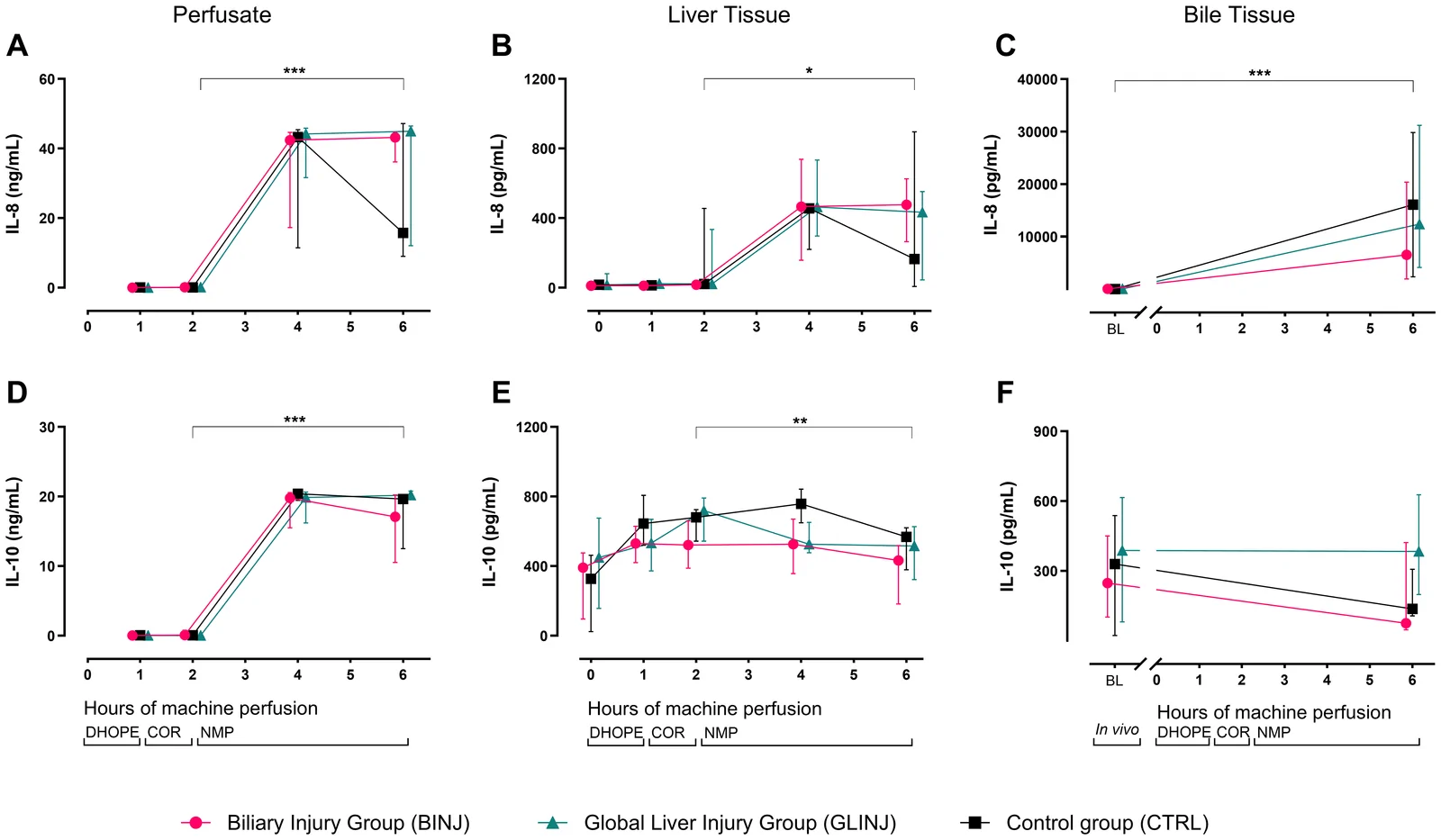
Pro- and anti-inflammatory cytokine concentrations in perfusate, liver, and bile tissue during liver machine perfusion using plasma-rich perfusate [**(A–C)**: IL-8, **(D–F)**: IL-10]. BL, baseline; DHOPE, dual oxygenated hypothermic perfusion; COR, controlled oxygenated rewarming; NMP, normothermic machine perfusion. Values are presented as median; vertical whiskers present interquartile range. Horizontal lines with *p*-values present end normothermic MP (6 h MP) compared to start normothermic MP (2 h MP; perfusate and liver tissue) and *in vivo* baseline (bile tissue), for CTRL alone or CTRL pooled with GlobalINJ and/or BileINJ when no significant difference between groups was identified. Colored asterisk present BileINJ or GlobalINJ, respectively, compared to CTRL over time during MP. *p < 0.05, **p < 0.01, ***p < 0.001.

Liver tissue cytokines IL-1β, IL-6, TNF, IL-8, and IL-10 did not differ between PRP groups and were thus combined in further analyses as described in Methods (**Figures 3B, E, H**, **4B, E**). All liver tissue cytokines, except for IL-10, increased significantly at end NMP (6 h MP) compared to start NMP (2 h MP, IL-1β: FC 6 [4–9], IL-6: FC 321 [141–739], TNF: FC 13 [6–22], IL-8: FC 20 [5–45], all *p* < 0.02, **Figures 3B, E, H**, **4B, E**). Liver tissue IL-10 significantly decreased at end NMP (6 h MP) compared to start NMP (2 h MP, FC 0.8 [0.6–1.0], *p* < 0.01, **Figure 4E**).

Bile tissue cytokines IL-6, TNF, IL-8, and IL-10 did not differ between PRP groups and were thus combined in further analyses as described in Methods (**Figures 3F, I**, **4C, F**). Bile tissue IL-6, TNF, and IL-8 significantly increased at end NMP (6 h MP) compared to baseline (IL-6: FC 19 [12–72], TNF: FC 22 [6–44], IL-8: FC 282 [105–564], all *p* < 0.001, **Figures 3F, I**, **4C**). Bile tissue IL-1β in the BileINJ group was significantly lower at end NMP (6 h MP) compared to CTRL (*p* = 0.001, **Figure 3C**), but increased similarly and significantly at end NMP (6 h MP) compared to baseline in all groups (all *p* < 0.002, **Figure 3C**).

#### 3.1.2 Complement activation in PPP versus PRP during MP

Perfusate, liver, and bile tissue sC5b-9 in PPP were significantly lower throughout MP compared to PRP (all *p* < 0.04, **Figures 5A–C**). Within the PPP group, sC5b-9 significantly increased in perfusate at end NMP (6 h MP) compared to start NMP (2 h MP, *p* < 0.05, **Figure 5A**).

**Figure 5:**
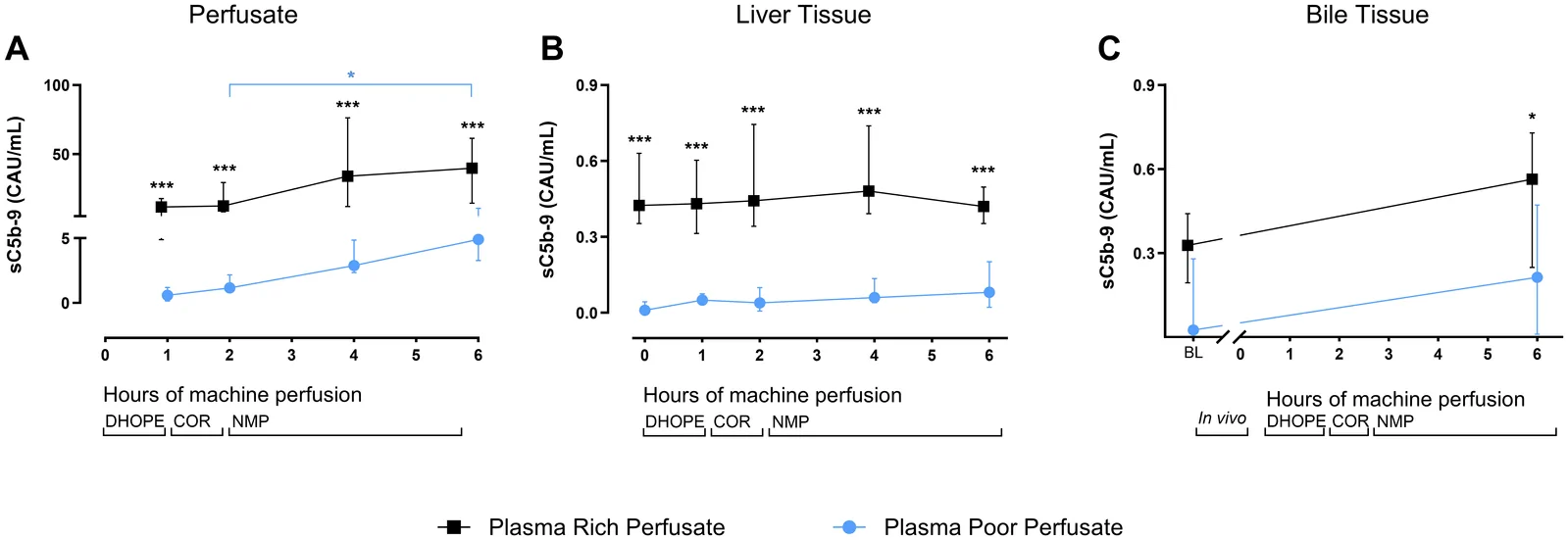
Complement activation (sC5b-9) in perfusate, liver, and bile tissue using plasma-rich and plasma-poor perfusate, respectively, during MP. BL, baseline; DHOPE, dual hypothermic oxygenated perfusion; COR, controlled oxygenated rewarming; NMP, normothermic machine perfusion. Values are presented as median; vertical whiskers present interquartile range with *p*-values presenting group with plasma-poor perfusate compared to plasma-rich perfusate at the set timepoint. Horizontal blue lines with *p*-values present end normothermic MP (6 h MP) compared to start normothermic MP (2 h MP; perfusate and liver tissue) and *in vivo* baseline (bile tissue) for the plasma-poor perfusate group. *p < 0.05, **p < 0.01, ***p < 0.001.

#### 3.1.3 Cytokine release in PPP versus PRP during MP

Perfusate cytokines IL-1β, IL-6, and IL-10 were significantly lower in PPP during MP (1, 4, or 6 h MP) compared to PRP (*p* < 0.05, **Figures 6A, D**, **7D**). TNF and IL-8 did not differ significantly between PPP and PRP (**Figures 6G**, **7A**). Within the PPP group, perfusate cytokines IL-1β, IL-6, IL-8, and IL-10 increased significantly at end NMP (6 h MP) compared to start NMP (2 h MP, all *p* < 0.04, **Figures 6A, D**, **7A, D**); TNF did not increase significantly during NMP (**Figure 6G**).

**Figure 6:**
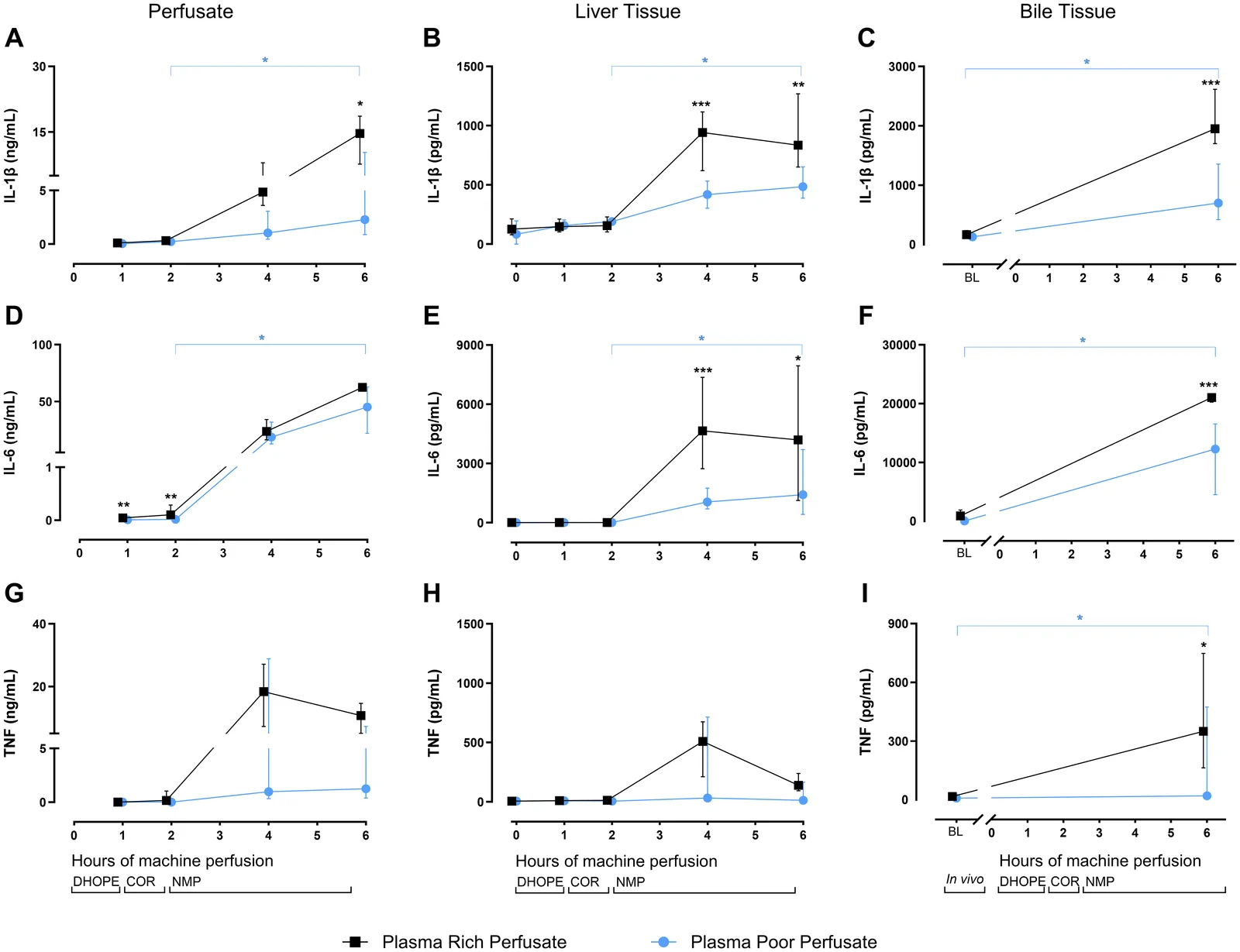
Proinflammatory cytokine concentrations in perfusate, liver, and bile tissue using plasma-rich and plasma-poor perfusate, respectively, during MP [**(A–C)**: IL-1β, **(D–F)**: IL-6, **(G–I)**: TNF]. BL, baseline; DHOPE, dual hypothermic oxygenated perfusion; COR, controlled oxygenated rewarming; NMP, normothermic machine perfusion. Values presented as median; whiskers present interquartile range with *p*-values presenting group with plasma-poor perfusate compared to plasma-rich perfusate at the set timepoint. Horizontal blue lines with *p*-values present end normothermic MP (6 h MP) compared to start normothermic MP (2 h MP; perfusate and liver tissue) and *in vivo* baseline (bile tissue), for the plasma-poor perfusate group. *p < 0.05, **p < 0.01, ***p < 0.001.

**Figure 7:**
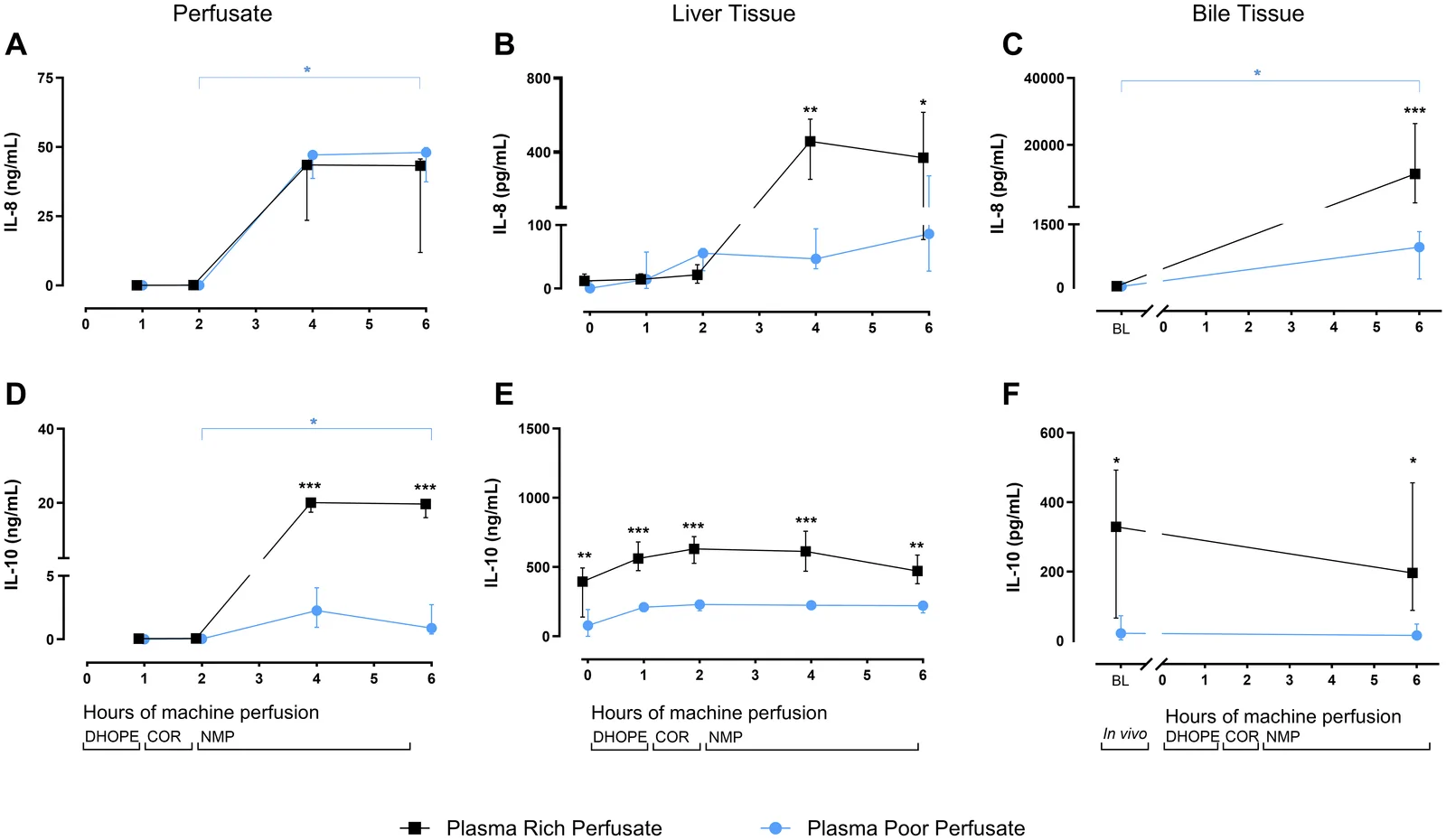
Pro- and anti-inflammatory cytokine concentrations in perfusate, liver and bile tissue using plasma-rich and plasma-poor perfusate, respectively, during MP [**(A–C)**: IL-8, **(D–F)**: IL-10]. BL, baseline; DHOPE, dual hypothermic oxygenated perfusion; COR, controlled oxygenated rewarming; NMP, normothermic machine perfusion. Values presented as median; whiskers present interquartile range with *p*-values presenting group with plasma-poor perfusate compared to plasma-rich perfusate at the set timepoint. Horizontal lines with *p*-values present end normothermic MP (6 h MP) compared to start normothermic MP (2 h MP; perfusate and liver tissue) and *in vivo* baseline (bile tissue), for the plasma-poor perfusate group. *p < 0.05, **p < 0.01, ***p < 0.001.

Liver tissue cytokines IL-1β, IL-6, IL-8, and IL-10 were significantly lower in PPP during MP (1, 2, 4, or 6 h MP) compared to PRP (all *p* < 0.04, **Figures 6B, E**, **7B, E**). TNF did not differ significantly between PPP and PRP (**Figure 6** h). Within the PPP group, liver tissue cytokines IL-1β and IL-6 increased significantly at end NMP (6 h MP) compared to start NMP (2 h MP, all *p* < 0.04, **Figures 6B, E**). Liver tissue cytokines IL-8 and IL-10 increased significantly when comparing start hypothermic MP (0 h MP) to end NMP (6 h MP, both *p* < 0.04).

Bile tissue cytokines IL-1β, IL-6, TNF, IL-8, and IL-10 were significantly lower in PPP at end NMP (6 h MP) compared to PRP (all *p* < 0.05, **Figures 6C, F, I**, **7C, F**). Within the PPP group, bile tissue cytokines IL-1β, IL-6, TNF, and IL-8 significantly increased at end NMP (6 h MP) compared to baseline (all *p* < 0.04, **Figures 6C, F, I**, **7C**). IL-10 did not increase significantly during NMP (**Figures 6I**, **7F**).

## 4 Discussion

In this study, we found that *ex situ* liver MP induced complement activation and cytokine release in perfusate, liver, and bile tissue regardless of induced ischemic injury prior to MP. Substitution of PRP with PPP showed substantially reduced complement activation and cytokine release. However, most mediators significantly increased in PPP as well.

With the exception of TNF in perfusate and IL-1β in bile tissue, the inflammatory pattern of general injury-independent complement activation and cytokine release observed in the PRP group is likely attributable to the inflammatory response elicited by IRI attributed to organ procurement (warm ischemia), preservation (cold ischemia), and finally MP itself, which all PRP livers have in common. However, we cannot exclude that longer MP duration might have unmasked immunological differences related to ischemic injury.

The terminal complement activation product sC5b-9 increased progressively during MP of porcine livers in perfusate and bile tissue, indicating sustained complement activation. While complement activation during liver graft reperfusion is well established—with sC5b-9 peaking in blood within the first hour post-reperfusion in human recipients (25–27)—our findings show a continuous rise throughout MP. This pattern aligns with previous reports of persistent complement activation during MP of both porcine and human kidneys (17, 18). The ongoing activation may, in part, result from the bio-incompatibility of the MP circuit, similar to what has been observed in extracorporeal systems such as cardiopulmonary bypass, extracorporeal membrane oxygenation, and hemodialysis (11–16). Exposure of blood to foreign surfaces and gas–blood interfaces can trigger complement activation, as confirmed in a kidney-free NMP model where complement components were activated solely by blood-to-material contact (18, 28). Terminal complement activation product sC5b-9 did not increase in the same way in liver tissue. This could be due to degradation of sC5b-9 under protein isolation or sC5b-9 remaining in the membrane and hence not being measured. However, C3a and downstream effects indicate a complement activation.

Perfusate C3a peaked at the start of rewarming, likely due to complement activation following whole blood filtration. The subsequent decrease could be explained by its short half-life (29). In contrast, C3a concentrations in microdialysate collected in hilar plate and bile increased during MP. Although not statistically significant, C3a trended to be higher in GlobalINJ (peak during rewarming) and in BileINJ (peak end rewarming) compared to CTRL. A previous study identified liver microdialysate C5a as a selective marker for ischemic events during liver graft reperfusion (30). Our findings indicate that microdialysate C3a should be investigated further as it may serve as a marker of ischemic liver and bile duct injury.

Our results show the robust release of both proinflammatory (IL-1β, IL-6, TNF, and IL-8) and anti-inflammatory (IL-10) cytokines into the perfusate, liver, and bile tissue during MP. Cytokine levels peaked between 2 and 4 h of NMP, consistent with previous observations in human (31–33) and porcine (34) liver and kidney (17, 18, 28) NMP models, highlighting that active metabolism is a prerequisite for cytokine production. The origin of cytokine release during liver MP remains largely unexplored. Cytokines are produced by various cells including immune cells (lymphocytes, macrophages, dendritic cells, mast cells, and monocytes) as well as endothelial, epithelial, and stromal cells (35). In our leukocyte- and platelet-depleted perfusate model, only liver-resident cells—such as Kupffer cells, hepatocytes, and biliary endothelial cells—are likely sources of cytokines (36, 37). The clinical consequences of cytokine release in MP perfusate, liver, and bile tissue warrants further investigation, especially in the aspect that the increase in cytokines in liver and bile tissue will follow into the recipient, as opposed to the cytokines in perfusate that will be flushed out before implantation.

Complement activation, represented by sC5b-9, was significantly higher in the PRP group compared to the PPP group during MP. The main source of complement proteins is plasma (38, 39). Thus, the increased presence of activation products in PRP is probably due to the increased availability of complement precursors in the plasma. Nonetheless, sC5b-9 levels in PPP also rose significantly at 4 h NMP compared to start of COR. Given that the liver synthesizes up to 90% of circulating complement proteins (38), this increase in the absence of plasma suggests the release of stored components as well as *de novo* hepatic synthesis and subsequent activation of complement proteins. This finding supports the concept of the liver being an active contributor to the inflammatory milieu during MP. Furthermore, despite washing the red blood cells according to this protocol, there will still be trace amounts of plasma proteins left in the supernatant; hence, some activation may be elicited by leftover plasma proteins. Nevertheless, these data support the use of PPP to limit initial complement activation, while also highlighting the liver’s intrinsic ability to generate complement proteins during perfusion.

In general, perfusate, liver, and bile tissue cytokines were substantially and significantly lower in PPP compared to PRP. Despite this general pattern of lower cytokine release in PPP, the cytokines still increased significantly during MP, except for TNF in plasma and liver tissue, and IL-10 in bile tissue. Notably, perfusate IL-6 and IL-8 increased with similar degree and kinetics to PRP, which might imply a non-complement-driven production/release of these specific cytokines in perfusate. The observations of reduced cytokine release in PPP are in line with a study demonstrating lower cytokine release in washed red blood cell perfusate compared to whole blood perfusate in porcine livers subjected to 24-h NMP (34). Taken together, our data support the use of PPP to limit cytokine release and modulate the inflammatory response upon reperfusion during NMP.

In our study, perfusate TNF is an exception from the remaining cytokines showing significantly lower perfusate levels in the ischemic injured GlobalINJ group compared to CTRL. This aligns with findings in a previous study on porcine livers subjected to 1 h of warm ischemia prior to procurement and subsequent 6 h of continuous NMP. This study showed distinct inflammatory responses and lower perfusate TNF during NMP in the warm ischemia livers compared to directly procured livers (33). These findings may imply that ischemic injury differentially alters the inflammatory response during liver MP with a stronger upregulation of the classical interleukins compared to the TNF/TNF-family pathway. This can be explained by TNF and IL receptors belonging to distinct receptor families with different signaling mechanisms (40, 41). Further research is needed to explore this TNF response.

Our study showed a significant inflammatory response in both injured and non-injured livers measured by complement activation and cytokine release. Removal of plasma from the perfusate had a significant effect on reducing the response; however, a statistically significant immunological response is still observed. The clinical consequences of such a response is not fully known, but a study of 30 human DBD and DCD livers transplanted after NMP found associations between perfusate IL-10 and intensive care stay, as well as a correlation between high IL-6 and graft loss (42). Another study reported higher perfusate IL-6 levels at the end of NMP in patients who died within 90 days after liver transplantation compared to survivors (39). An MP study on porcine kidneys found higher activation of complement associated with impaired renal function and a strong correlation between complement activation and cytokine release of IL-6 and IL-8 (17). These data suggest that complement and cytokines could be candidate biomarkers assisting in identifying unhealthy livers and monitoring negative effects of the treatment process, and that managing substances that activate the innate immune system is key to ensuring a successful outcome.

Interventions specifically targeting innate immune activation are warranted, as complement and inflammatory cytokines remain profoundly elevated in our PPP group as well. Dual inhibition of complement component C3 or C5 combined with the neutralization of the CD14 molecule, a co-receptor for several TLR receptors including TLR4 and TLR2, has been proposed to be a clinically relevant treatment to block an inappropriate and overwhelming upstream inflammatory reaction and restore homeostasis (43). This has been described in sepsis models (44–46) and has recently also been proven in sterile ischemia–reperfusion settings. Here, C5 and CD14 inhibition efficiently prevented systemic and local inflammation and improved organ dysfunction in a brain death mouse model (44) and a porcine polytrauma model (45). To date, no conclusion can be drawn about long-term effects of complement activation and cytokine release in the liver MP setting, and thus, interventional studies should be conducted ideally in an MP setting of human livers. However, the porcine model is the animal model closest to human physiology, and anatomy and previous reviews support the porcine liver MP model’s data transferability to human livers (46). Our model proves that inflammation occurs during MP in pigs comparable to humans, enabling the preclinical testing of dual inhibition with the aim of translation in future studies.

This study has limitations. While the power calculation adhered to animal welfare regulations, the small number of animals limits generalization and may also explain the missing differences in inflammatory responses between ischemic injury groups and CTRL we otherwise found when investigating metabolism, viability, liver function tests, and dielectric properties of the same livers (47). However, we can conclude that *ex situ* NMP is a strong activator of the complement system and leads to cytokine release. This is supported by our findings of strong complement activation in both PRP and PPP groups, most likely due to the initial impact of MP itself in combination with an unavoidable impact of organ reperfusion. This strong complement activation occurring in all groups may also be the reason for not being able to distinguish the ischemic injury from control groups. This study exclusively evaluated presumably healthy livers, which differ from human donors with wide-ranging medical histories, especially fatty or cirrhotic livers. Nevertheless, ischemic injuries induced in our study may relate to livers obtained from donation after cardiac death or after prolonged storage (global injury group) or represent circulatory disturbances (bile injury group). All livers were subject to 1–2 h of cold storage after procurement due to experimental logistics including transport of the liver from the animal to the MP facility and cool down of the perfusion machine to 12°C. Ideally, the livers would have been placed directly from procurement to MP without intermediate cold storage. However, this was logistically not possible. As all livers were subject to this same duration of SCS, it is not considered to affect differences between groups.

## 5 Conclusion

Immunological analysis of complement activation products and downstream cytokine release revealed innate immune system activation during MP of livers both with and without induced ischemic injury. Plasma in the perfusate determines the magnitude of inflammation, although PPP still leads to inflammation, implying a pivotal role of the liver itself in reperfusion injury. PPP should be used in future studies, and further interventions on the innate immune system should be investigated to reduce the hazardous effects of reperfusion injury with the aim of increasing the number of transplantable organs.

## Data Availability

The raw data supporting the conclusions of this article will be made available by the authors, without undue reservation.
