# Supplementary material for: Immune activation during liver machine perfusion is shaped by perfusate composition and is independent of the ischemic injury
Source: Front Immunol. 2026 Sep 14;17:1874888. doi: 10.3389/fimmu.2026.1874888 (PMC13616650; doi:10.3389/fimmu.2026.1874888)
Supplement: Supplementary file 1 [file SupplementaryFile1.docx]

Supplementary Material

**Supplementary Figure 1.** Complement and cytokines in the autologous plasma *in vivo* at baseline and prior procurement and *ex situ* during machine MP using plasma-rich and plasma-poor perfusate. (Panel A: sC5b-9, Panels B-F: cytokines IL-1β, IL-6, TNF, IL-8, IL-10). No significant differences in the autologous plasma were seen between the groups prior to MP, all p<0.05. MP - machine perfusion; PRP- Plasma-rich perfusate; PPP- Plasma-poor perfusate. BL- baseline; DHOPE - dual hypothermic oxygenated perfusion; COR - controlled oxygenated rewarming; NMP – normothermic machine perfusion

**Supplementary Figure 2.** Complement activation was assessed in the process of leukocyte and platelet filtration of whole blood and in the process of plasma washout. A) Leukocyte and platelet depletion of porcine whole blood significantly increased complement activation (sC5b-9) compared to non-filtrated whole blood (p<0.002) B) Plasma washout in the process to plasma-poor perfusate reduced sC5b-9 levels to baseline levels. Plasma-rich perfusate (PRP, Plasma-poor perfusate (PPP), Leukocyte and platelet depleted whole blood (LPDWB).
