# Supplementary material for: Immune activation during liver machine perfusion is shaped by perfusate composition and is independent of the ischemic injury
Source: Front Immunol. 2026 Sep 14;17:1874888. doi: 10.3389/fimmu.2026.1874888 (PMC13616650; doi:10.3389/fimmu.2026.1874888)
Supplement: Supplementary file 2 [file Table1.docx]

Supplementary Table

**Supplementary table 1**

(A) Composition of perfusion solution used during COR and NMP in PRP

| **Components of perfusion solution at start COR and NMP** | **Quantity at start COR** | **Components further added to obtain subnormal-normal values during COR and NMP** |
| --- | --- | --- |
| Leukocyte and platelet depleted porcine whole blood (RBC and plasma) | >1500mL | add when Hb<5.0g/dL |
| Humane Albumin 200g/L | 100 mL |  |
| Modified parental nutrition (modTPN) | 20 mL |  |
| Multivitamins for infusion (Cernevit) | 2 mL |  |
| Concentrated trace elements (Addaven) | 10 mL |  |
| Humalog 100 U/ml | 1 mL, further added when Gluc > 12.0 mmol/L | Add 100 U Humalog if blood glucose > 12.0 mmol/L |
| Calcium glubionat 10% | 0-20 mL (10) | Add 5-10 mL when Ca^2+^ < 0.9 mmol/L |
| Sodium bicarbonate 4.2% | 62 mL, further added when pH < 7.2 | Add 20mL 4.2% NaHCO_3_ to reach pH > 7.2 |
| Heparin 5000 IE/ml | 0.5 mL at start. 0.2 mL every hour | Add 0.2 ml every hour during NMP |
| Glucose 50% | When needed for Gluc > 4.0 mmol/L | Add when Glucose < 4.0 mmol/L |
| Potassium Chloride | When needed for K > 3.0 mmol/L | Add when K < 3.0 mmol/L |
| Ringer acetate | When needed for Hct ~ 20% | Add when Hematocrit > 20% |

(B) Composition of perfusion solution used during COR and NMP in PPP

| **Components of perfusion solution at start COR and NMP** | **Quantity at start COR** | **Components further added to obtain subnormal-normal values during COR and NMP** |
| --- | --- | --- |
| Plasma free leukocyte and platelet depleted porcine whole blood, (RBC) | 500 mL |  |
| Voluven 6% | 300 mL |  |
| Humane Albumin 200 g/L | 200 mL |  |
| Modified parental nutrition (modTPN) | 20 mL |  |
| Multivitamins for infusion (Cernevit) | 2 mL |  |
| Concentrated trace elements (Addaven) | 10 mL |  |
| Ringer acetate (mixed with RBC) | 250 mL |  |
| Humalog 100 U/ml | 1 mL, further added when Gluc > 12.0 mmol/L | Add 100 U Humalog if blood glucose > 12.0 mmol/L |
| Calcium glubionat 10% | 10 mL | Add 5-10 mL when Ca^2+^ < 0.9 mmol/L |
| Sodium bicarbonate 4.2% | 110 mL, further added when pH < 7.2 | Add 20mL 4.2% NaHCO_3_ to reach pH > 7.2 |
| Heparin 5000 IE/ml | 2.0 mL at start. 0.2 mL every hour | Add 0.2 ml every hour during NMP |
| Glucose 50% | When needed for Gluc > 4.0 mmol/L | Add when Glucose < 4.0 mmol/L |
| Potassium Chloride | When needed for K > 3.0 mmol/L | Add when K < 3.0 mmol/L |
| Ringer acetate | When needed for Hct ~ 20% | Add when Hematocrit > 20% |

PRP- Plasma Rich Perfusate; PPP- Plasma Poor Perfusate; COR - controlled oxygenated rewarming; NMP – normothermic machine perfusion; RBC – red blood cells

Note: Composition of the perfusion solution is designed to provide a fluid with physiological colloid osmotic pressure and osmolality based on the Groningen DHOPE-COR-NMP protocol (1, 2). It consists of a mix of albumin, modified parenteral nutrition, multivitamins for infusion, concentrated trace elements, fast-acting insulin, calcium, sodium bicarbonate, heparin and RBC as oxygen carrier. Components will be added to the system during machine perfusion to obtain subnormal-normal values depending on pH, blood glucose and electrolytes.

References: ^1.^van Leeuwen O. DHOPE – COR – NMP with RBCs Protocol for organ perfusionists. 2020. ^2.^de Vries Y. WERKPROTOCOL ,DHOPE – COR – NMP Trial Onderzoeker. 2020.
